# An Investigation into a Miniature Saltless Solar Pond

**DOI:** 10.3390/ma15175974

**Published:** 2022-08-29

**Authors:** Mervette El-Batouti, Mona M. Naim, Nouf F. Al Harby, Mahmoud M. Elewa

**Affiliations:** 1 Chemistry Department, Faculty of Science, Alexandria University, Alexandria 21526, Egypt; 2 Chemical Engineering Department, Faculty of Engineering, Alexandria University, Alexandria 21526, Egypt; 3 Department of Chemistry, College of Science, Qassim University, Qassim 52571, Saudi Arabia; 4 Arab Academy for Science, Technology and Maritime Transport, Alexandria P.O. Box 1029, Egypt

**Keywords:** saltless non-convective solar pond, long-term solar energy storage, convection currents, thermal energy, cruciform baffles, corrosion

## Abstract

A simple, miniature saltless Solar Pond (SP) was designed and constructed in the present work. It consisted of a Plexiglas container with a square cross-section, within which cruciform baffles were suspended in the upper half of the pond, and copper coil tubing was fitted in the middle of the lower zone to function as a heat exchanger without disturbing the pond’s inertia. Different variables’ effect on the water’s temperature rise at various vertical locations within the pond were investigated. These variables included the presence of the cruciform baffles, the inclination of a mirror fixed to the top rim of the pond, a glass transparent cover (GC), and the presence or absence of a gel thickening material to increase the water viscosity inside the SP, the climatic conditions, and the presence of glass wool (GW) in the lower section of the SP. For an experiment, an estimated energy balance was performed, and the thermal storage efficiency was calculated. The best obtained thermal storage efficiency was 32.58% in the presence of the cruciform baffles, a 45° inclination of a mirror fixed to the top rim of the SP, at an ambient temperature of 30 °C on a calm, sunny day with a wind speed of 7 km/h.

## 1. Introduction

Solar energy is the world’s most abundant permanent source of energy. The amount of solar energy intercepted by the planet Earth is 170 trillion kW, an amount 5000 times greater than the sum of all other inputs (terrestrial, nuclear, geothermal, gravitational energies and lunar gravitational energy). The amount of the sun’s energy intercepted by the earth is only a tiny fraction—one-thousandth of one million—of the total energy released by the conversion of four million tons of hydrogen per second to helium in the sun. Solar energy impinging on the earth’s atmosphere is dilute (approximately 430 BTU/ft^2^ h) and is of two forms: direct radiation and diffuse radiation. Direct radiation is collimated and capable of casting a shadow, while diffuse radiation is dispersed, or reflected by the atmosphere, and not collimated. The ratio of direct to diffuse radiation varies with time and location so, while it is in the order of 5 in a small town, for a large city it may only be in the order of 2 [1].

Solar energy is available in the daytime at no cost; however, it is absent at night. Being intermittent, it needs to be stored, both at night-time and in inclement weather. Therefore, a means of storing solar heat energy has to be implemented to enable its use either throughout the day or in winter. To this end, solar energy storage has been investigated in different ways, of which sensible heat and/or latent heat are used for short-term heat storage, while salty SPs are used to store heat in summer for later use in winter [2,3].

Solar ponds (SPs) are non-conventional thermal energy sources [4]. They are large-scale energy accumulators which supply thermal energy. They can be used for various applications such as water heating [5], air heating [6], water desalination [7,8], refrigeration and air conditioning [9,10,11], and power generation [12,13]. They work by creating a difference in salt density in the bottom layer of water. This limits the natural movement of water between layers and the transfer of heat from the top layer of water to the air. Various salts are employed to construct the salt gradient in the solar pond’s bottom layer, thus maintaining three layers: the upper convective zone (UCZ), the nonconvective zone (NCZ), and the lower convective zone (LCZ), in which the salinity is proportionate to the depth. The high-density salt containing LCZ is used as a storage medium. Prior research on solar ponds focused on altering the pond’s solution and concentrations. As the pond solutions, sodium carbonate, sodium chloride, magnesium chloride, and urea were used. The capture or storage of solar radiant heat is entirely passive [14,15].

The most documented version of the solar pond is the salt-gradient solar pond (SGSP), wherein the increase in the salt concentration with the depth prevents convection in the upper region. Solar ponds offer numerous advantages to their users, including low operation costs, simple construction, enhanced stability, a large thermal storage capacity, fewer maintenance requirements, a shorter construction time, no need for highly skilled labour to construct or operate, being environmentally friendly, and producing a large amount of heat with less evaporation. Therefore, they are the optimal choice for applications requiring low-grade energy [16]. SGSPs have been subjected to several theoretical and experimental investigations all over the world [17,18,19]. The general problems associated with SGSPs are (i) the establishment and maintenance of the salt gradient, (ii) the destabilisation of the salt gradient by wind-induced brine agitation and evaporation from the UCZ, (iii) the destabilisation effect of the heat removal on the salt gradient, (iv) the high cost of salt in some areas, (v) the maintenance of brine clarity, (vi) the environmental effects of potential brine leakage on the soil and groundwater, and (vii) the corrosive properties of the brine [20,21].

Saltless, nonconvective SPs with various stabilising mechanisms and/or fluid media for convection suppression have been offered as a solution to the SGSPs’ fundamental issues. Hull [22] proposed the membrane-stratified solar pond idea, in which closely spaced transparent membranes are positioned horizontally or vertically in the upper section of the pond fluid to quench the convective heat transmission. Shaffer [23] was the first to investigate the use of a polymer gel as an insulating layer. Wilkins et al. [24] then conducted an experimental analysis of polymer gels floating in pond waters. Ortabasi et al. [25] presented honeycomb-based, saltless, nonconvective SPs based on convection suppression devices developed for flat plate collectors. Ortabasi et al. [25] described an open honeycomb structure with a small layer of oil floating on the surface of hot water. However, field studies with the aforementioned technology [26,27] revealed that an open honeycomb acquires dirt and soon loses its transmittance, and hot air escapes from the honeycomb cells. As a result, the arrangement including a honeycomb with a thin transparent cover at the top and a thin transparent sheet or oil layer at the bottom seems to be the most appropriate. Sharma and Kaushika [27] investigated the thermal performance of such a system, theoretically. Experimental and theoretical results, for a small saltless SP with three semi-transparent, air-filled surface insulation layers, were presented by Kaminto et al. [28].

Currently, the research is conducted in an SP using nanofluids [29,30,31,32,33] and phase change materials PCMs [34,35]. Copper ethylamine was used to inhibit the formation of algae in the SP. In solar pond research, structural modification has recently gained pace. As structural changes, trapezoidal, rectangular, and U-shaped ponds with an exterior heat exchanger were used. The solar pond with the trapezoidal construction had a greater temperature than the rectangular structure. A rotating cover was employed in a solar pond during the day as a reflector, and at night as an insulator. Due to the larger heat capacity, plastic glazing, ball bearings, and pebbles were incorporated into an SP to boost the temperature of the LCZ [36]. PCMs [34,37], nanofluids [38,39], and expanded fins are the most cost-effective ways to improve the thermal performance of solar ponds [40].

Applications of solar energy are diverse. However, despite its free availability to all, it is discontinuous. Accordingly, some means for capturing and storing this energy must be done in one way or another, of which solar ponds (SPs) provide a thermal storage capacity spanning the seasons. In view of this, the present investigation deals with the performance of a miniature, saltless SP of a specific and uncomplicated design to explore the effects of such as the presence or not of baffles; the presence of GC; the slope of the mirror at the top of the SP; the presence of GW inside the SP and its quantity; the presence of a blackened wooden base and black rubber sheet; the solar radiation intensity; and the presence of wind.

## 2. Experimental Section

The present work uses a miniature, saltless 15 × 15 × 40 cm SP of a straightforward design, made of Plexiglas. The greater depth at the bottom layer often results in a darkening that absorbs a great deal of solar radiation [41,42,43] and, into this, cruciform baffles are fitted to suppress the convection currents in the UNCZ; this was investigated for its ability in storing solar heat. The variables to be investigated for their effect on the water temperature, at different locations in the pond as a function of time of exposure to solar radiation, include the number of baffles, the presence of insulation around the pond, the number of sides insulated with black wool, the presence of glass wool (GW) or a felt pad inside the SP, the presence of a mirror at the top rim of the pond and its horizontal inclination, the effect of climatic conditions, the presence of a gel in the lower convective zone (LCZ) as a means of increasing the water viscosity, and the presence of GW in the UNCZ and its quantity. The dates of the experiments were recorded and the approximate values of the solar insolation were obtained from the insolation tables by Kreider and Kreith [44], at 32° N latitude, which is the value of solar radiation falling on a horizontal surface, in Alexandria, Egypt. 

An energy balance will be done to find out how much heat is lost under different conditions. For some experiments, the efficiency of the solar energy storage will be calculated, and then the best conditions will be given. Results shall be presented in figure form-relating temperatures at various locations in the SP versus the time of day.

### 2.1. Materials

Plexiglas sheets were used for the construction of the SP. A glass cover (GC) with a thickness of 1 mm provided a top cover for the SP to prevent surface evaporation and heat loss, concomitantly allowing the solar radiation to pass through. A blackened wooden base provided insulation for the bottom of the pond. A square mirror placed at the top rim of the pond was used to reflect the solar radiation to the top surface of the pond. A black rubber sheet was introduced inside the pond to cover the bottom layer. A copper coil was fitted in the middle of the lower half of the pond, and a 200 W pump forces water through the coil to exchange heat from the LCZ, to be used for external heating. Polyethylene tubing connecting the coil and external water in a beaker permitted the circulation of water through the coil, whereby the water is heated. Glass wool is an insulating material made from fibres of glass arranged using a binder into a texture like wool. The process traps many small pockets of air between the glass fibres, and these small air pockets result in high thermal insulation properties. GW was used in an effort to dampen the convection currents in the pond while providing continuous channels of water to allow heat conduction.

### 2.2. Methods

**Figure 1:**
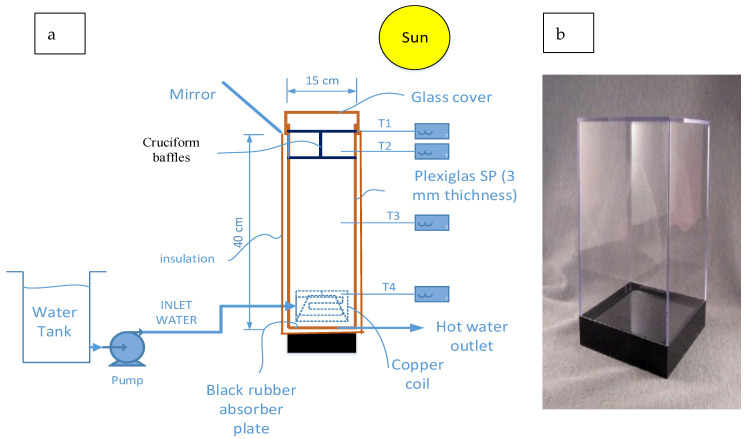
(**a**) shows a schematic diagram of the set-up; (**b**) a photograph of SP before assembly.

The SP consisted of a 3 mm thick Plexiglas container, 40 cm high and of square form, with a 15 × 15 cm cross-section. To limit heat loss, the SP could be manufactured from a specific, low-emission plastic material having solar radiation qualities comparable to those of glass [45]. Inside the container, copper coil tubing was fitted with an inlet and an outlet for water, which functioned as a heat exchanger for the heat collected at the bottom of the pond without disturbing the inertia of the water contained in the pond. The top half-section of the container (UNCZ) was fitted with Plexiglas partitions of a square cross-section (cruciform baffles), which extended from one side to that opposite it, in order to minimise heat loss by convection currents from the top and sides of the SP. In this way, the square cross-section was divided into four squares (compartments) in the top half of the container. In some experiments, other partitions of cruciform shape are to be later investigated for their effect in further damping convection currents, making the UNCZ divided into 16 squares. A square, block of wood, painted matte black, functioned as a base for the SP to avoid heat loss from the bottom of the SP to the ground. Additionally, a square black rubber sheet obtained from waste automobile tires was placed on the bottom of the SP to function as insulation. Figure 1 shows a schematic diagram of the set-up, comprising the cruciform baffles, plus the pump, copper coil and a container of water, which will be allowed to recycle through the copper coil, through which the heat is drawn and used to heat the external water. The diagram also shows the position of four different thermometers placed as follows: T1, which measures the temperature at the top of the pond, T2, which gives the temperature midway between the height of the cruciform baffles, and T3 and T4, which measure the temperatures just below the baffles, and at the centre of the coil, respectively.

### 2.3. Procedure

The SP was filled with water to the top rim, after fitting the baffles in place in the UNCZ, and then the SP was hermetically sealed by tightly covering it with the GC. The four temperatures were recorded versus time of day, at hourly intervals. The times of the experiments were somewhat different from each other, but, in general, they varied between 3–6.5 h, around noon.

### 2.4. Variables Investigated

Different variables were investigated for their effect on the temperature rise of the water at the different locations in the SP, as well as the temperature in the external beaker, and these were (1) the presence or absence of the baffles, (2) the presence of the GC, (3) the slope of the mirror at the top of the SP, (4) the presence of the GW inside the SP and its quantity, (5) the presence of the blackened wooden base and black rubber sheet, (6) the solar radiation intensity and presence of wind, and (7) the presence of the tubing connecting the copper coil and the external water in the beaker.

### 2.5. Computation of Thermal Efficiency of the SP

To compute the average efficiency of the SP performance, an approximate energy balance will be performed. The thermal efficiency of the SP is defined as follows:

(1) $$\mathrm{Thermal}\;\mathrm{efficiency}\;\mathrm{of}\;\mathrm{the}\;\mathrm{solar}\;\mathrm{pond}=\frac{T_{LCZ}-T_{a}}{T_{LCZ}}\times 100$$

where: $T_{LCZ}$ = temperature stored in LCZ, °C; $T_{a}=$ ambient temperature, °C.

Ignoring the temperature gradient within the SP, the energy balance in the UNCZ, in which the heat is assumed to be transferred only by conduction, is

(2) $$Q_{stored}=Q_{in}-Q_{out}=V\rho C_{p}\frac{dT}{dt}$$

where: $Q_{stored}$ = the net heat stored in the pond (W); $Q_{in}$ = the heat transfer to the pond (W); $Q_{out}$ = the heat transfer from the pond (W); *V* = the pond volume (m^3^); *ρ* = the density of the pond water (Kg/m^3^); $C_{p}$ = the specific heat of the pond water (J/kg. K); $\frac{dT}{dt}$ = the rate of change of temperature of the pond water (K/s).

The right-hand side of Equation (2) may be replaced by another term, as shown in Equation (3)

(3) $$V\rho C_{p}\frac{dT}{dt}=\left(Q_{solar}\right)-\left(Q_{ev.}+Q_{r.w-a}+Q_{c.w-a}+Q_{su}\right)$$

where: $Q_{solar}$ = the solar radiant heat gain to SP (W); $Q_{ev.}$ = the heat transfer due to evaporation at the pond surface (W); $Q_{r.w-a}$ = the thermal radiant heat transfer from the water to the ambient air (W); $Q_{c.w-a}$ = the convective heat transfer from the water to the ambient air (W).

However, $Q_{ev.}$ may be ignored due to the surface being covered with the GC. In addition, $Q_{r.w-a}$ may also ignored due to its small value, and $Q_{c.w-a}$ as well, since the baffles are placed to suppress convective heat loss. Based on the energy consumption, the transient behaviours of the heat transfer were developed for the two zones of the SP. Some assumptions, however, were made to simplify the thermal analysis.

(4) $$\;A_{solar}=\left(I_{b}\tau_{rb}\tau_{ab}+I_{d}\tau_{rd}\tau_{ad}\right)\;A_{pond}$$

where: $I_{b}$ = the beam radiation (4694 W/m^2^); $I_{d}$ = the diffuse radiation (W/m^2^); $\tau_{rb}$ = the transmissivity based on the reflectance of the beam radiation; $\tau_{ab}$ = the transmissivity based on the absorption of the beam radiation; $\tau_{rd}$ = the transmissivity based on reflectance of the diffuse radiation; $\tau_{ad}$ = the transmissivity based on the absorption of the diffuse radiation; $A_{pond}$ = the surface pond area of 0.0929 m^2^.

Assuming the diffuse to beam radiation equals 1:5 [44], due to the experimental site being in a spacious and uncrowded calm region $\tau_{rb}$, $\tau_{ab}$, and $\tau_{rd}$ are ignored due to their negligible values.

(5) $$Q_{ev.}=\frac{Lh_{conv}A_{pond}\left(P_{v}-P_{\infty }\right)}{1.6C_{p}P_{atm}}$$

where: L = the latent heat of evaporation of the pond water (J/kg); $h_{conv}$ = the convection heat transfer coefficient (W/m^2^ K), $P_{atm}$ = the atmospheric pressure (bar); $P_{v}$ = the saturation vapour pressure corresponding to the surface water temperature (*P*_∞_) (Ignored).

(6) $$h_{conv}=5.7+3.8v$$

where: v = the average monthly wind speed

(7) $$P_{v}=exp\left(18.403-\frac{3885}{T_{u}-230}\right).$$

where: $T_{u}$ = the temperature of UCZ (K).

(8) $$P_{\infty }=R_{h}\;exp\left(18.403-\frac{3885}{T_{amb}+230}\right)$$

where: $R_{h}$ = the monthly average relative humidity (%); $T_{amb}$ = the ambient temperature (K); $P_{\infty }$ = the partial pressure of the water vapour in the air (atm).

(9) $$Q_{r.w-a}=\varepsilon_{w}\sigma A_{pond}\left(T_{u}^{4}-T_{sky}^{4}\right)$$

where: $\varepsilon_{w}$ = the emissivity of the pond water (about 0.3); σ = the Stefan-Boltzman’s constant (3.102 × 10^−19^ BTU/h ft^2^ °C^4^).

The sky temperature is given by Kurt et al. [46] as:

Assuming $T_{sky}$ = 20 °F = −6.66 °C [44] and the average $T_{u}$ = 40 °C ($Q_{r}$ = 576,320.289 × 10^−19^ BTU/h)

(10) $$T_{sky}=T_{amb}-{\left(0.55+0.061\sqrt{P_{\infty }}\right)}^{0.25}$$

(11) $$Q_{su}=K_{sw}A_{pond}\frac{dT}{dx}$$

where: $K_{sw}$ = the side walls’ thermal conductivity (W/m K); $\frac{dT}{dx}\;$= the temperature gradient through the side walls (K/m).

Thus, the heat balance can be written as:

(12) $$\begin{array}{cc} V\rho C_{p}\frac{dT}{dt} & =A_{pond}(\left(I_{b}\sigma_{rb}\sigma_{ab}+I_{d}\tau_{rd}\tau_{ad}\right)-\left(\frac{Lh_{conv}\left(P_{v}-P_{\infty }\right)}{1.6C_{p}P_{atm}}\right)\\ & -\left(\varepsilon_{w}\sigma \left(T_{u}^{4}-T_{sky}^{4}\right)\right)-\left(h_{conv}\left(T_{u}-\left(T_{amb}\right)\right)-\left(K_{sw}\frac{dT}{dx}\right)\right)) \end{array}$$

(13) $$Q_{stored,UCZ}=Q_{stored,UCZ}-\left(Q_{ev.}+Q_{r.w-a}+Q_{c.w-a}+Q_{sw,UCZ}\right)$$

where: $Q_{stored,UCZ}$ = the net heat stored energy in the UCZ (W); $Q_{ev.}$ = the heat transfer due to evaporation at the pond surface (W); $Q_{c.w-a}$ = the thermal radiant heat transfer from the water to the ambient air (W).; $Q_{c.w-a}$ = the convective heat transfer from the water to the ambient air (W); $Q_{sw,UCZ}$ = the conductive heat loss from the sidewalls of the UCZ (W); $Q_{solar,UCZ}$ = the solar radiant heat gain to the UCZ (W).

(14) $$Q_{solar,UCZ}=A_{UCZ}{\left(I_{b}\tau_{rb}\tau_{ab}+I_{d}\tau_{rd}\sigma_{ad}\right)}_{Z=\left(X\_1-\delta \right)}$$

where: Z = the depth of the pond which transmits the solar energy to be calculated (m); *δ* = the thickness where the long-wave solar energy is absorbed (m); $X_{1}$ = the thickness of the UCZ (m).

(15) $$\begin{array}{cc} Q_{stores,UCZ} & =A_{UCZ}(A_{UCZ}{\left(I_{b}\tau_{rb}\tau_{ab}+I_{d}\tau_{rd}\sigma_{ad}\right)}_{Z=\left(X_{1}-\delta \right)}-\left(\frac{Lh_{conv}\left(P_{v}-P_{\infty }\right)}{1.6C_{p}P_{atm}}\right)-\left(\varepsilon_{w}\sigma_{}\left(T_{UCZ}^{4}-T_{sky}^{4}\right)\right)\\ & -\left(h_{conv}\left(T_{UCZ}-T_{amb}\right)\right)-\left(K_{sw}\frac{\left(T_{UCZ}-T_{amb}\right)}{\Delta t}\right)) \end{array}$$

where: Δt = the thickness of side and bottom walls (m).

For the energy balance for the heat storage zone (HSZ), the heat in this zone can be written as follows:

(16) $$Q_{stored,\;\;HSZ}=Q_{solar,\;HSZ}-\left(Q_{1,HSZ}+Q_{sw,HSZ}+Q_{bottom}\right)$$

where: $Q_{stored,\;HSZ}$ = the net heat stored energy in the HSZ (W); $Q_{solar,\;HSZ}$ = the amount of solar radiant heat entering the HSZ, which is transmitted from the NCZ (W); $Q_{1,HSZ}\;$= the conductive heat loss from the NCZ to the UCZ (W); $Q_{sw,HSZ}$ = the conductive heat loss from the side walls of the HSZ (W); $Q_{bottom}$ = the conductive heat loss from the bottom of the HSZ (W).

In which the conductive heat loss from the bottom of this zone is:

(17) $$Q_{bottom}=K_{f}\frac{\left(T_{HSZ}-T_{ground}\right)}{\Delta t}$$

The heat balance equation for the HSZ can be written as:

(18) $$\begin{array}{cc} Q_{stored,\;HSZ} & =A_{HSZ}({\left(I_{b}\tau_{rb}\tau_{ab}+I_{d}\tau_{rd}\sigma_{ad}\right)}_{Z=\left(X_{3}-\delta \right)}-\left(K_{f}\frac{\left(T_{HSZ}-T_{NCZ}\right)}{\Delta x_{3}}\right)\\ & -\left(K_{sw}\frac{\left(T_{HSZ}-T_{amb}\right)}{\Delta t}\right)-\left(K_{fbottom}\frac{\left(T_{HSZ}-T_{ground}\right)}{\Delta t}\right)) \end{array}$$

### 2.6. Uncertainty Analysis

Using PT 100 temperature sensors with an accuracy of 0.5 °C, the temperature distribution of the SP’s various zones was determined. With an accuracy of 0.1 °C, these temperature sensors were connected to a data taker system for measuring the temperature in the various zones. Using the root sum of squares methods [47], the overall measurement uncertainty was determined [48].

(19) $$\Delta_{total}=\sqrt{{\left(\Delta_{instrument}\right)}^{2}+{\left(\Delta_{sensor}\right)}^{2}}$$

where: $\Delta_{instrument}$ = 0.1 °C, $\Delta_{sensor}$ = $\frac{0.5\;{}^{\circ}C}{\sqrt{3}}=0.28\;{}^{\circ}C$, then $\Delta_{total}$ = 0.38 °C.

## 3. Results and Discussions

The effect of the different variables presented in Table 1 that could affect the performance of the SP is discussed below.

### 3.1. Effect of Mirror Slop

**Figure 2:**
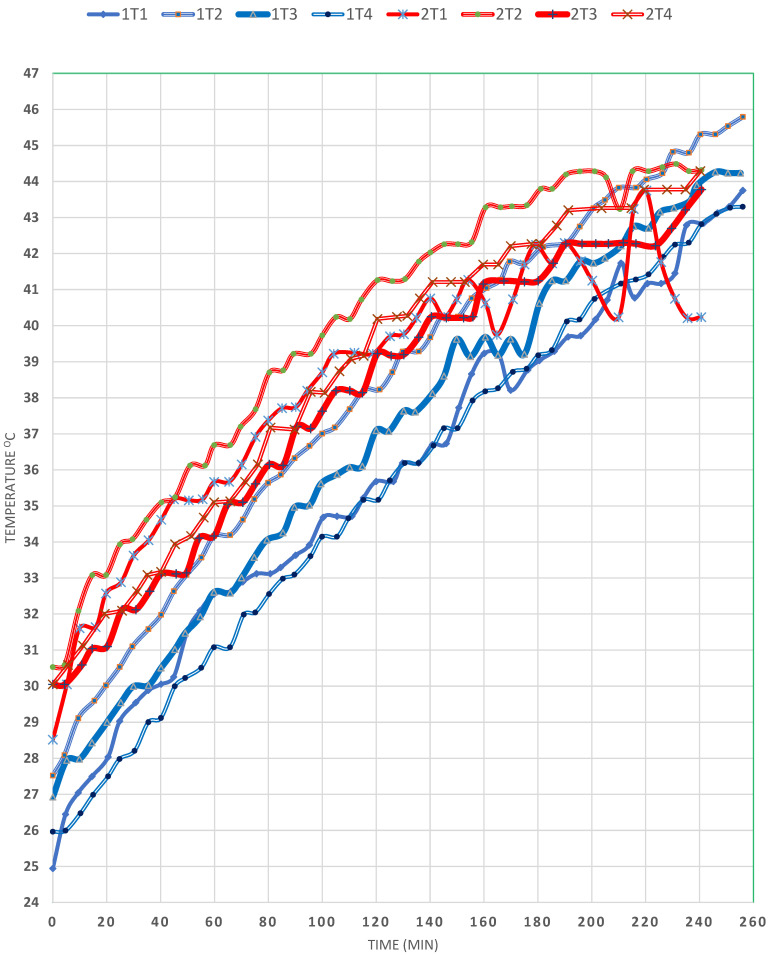
Effect of the mirror slope on the temperature profiles in the SP (EXPs (1) and (2)).

Comparing EXP (1) and (2), shown in Figure 2, in which the horizontal mirror tilt was 80° and 45°, respectively, and T_a_ was 31.5° and 30°, respectively, and the two experiments were done at the same time of day, it can be seen that T1 reached 42 °C and 40 °C, respectively, after 300 min. This proves that a slope equal to 45°, at this particular time of day at which the solar radiation is around its maximum, results in the maximum reflection of solar radiation onto the surface of the SP, since the angle of incidence onto the mirror equals the angle of reflection onto the water surface. However, it is observed that, in EXP (2), the initial temperature was below that in EXP (1), meaning that more radiation was obtained at the higher slope. Moreover, it was noted that, in the case of EXP (2), the temperature of T4 was higher, the highest among all the other temperatures measured, followed by that of T3_,_ located just below the baffles, which is attributed to the effective damping effect on the convection currents by the cruciform baffles, which assist in better solar heat storage. On the other hand, in EXP (1), the difference between the temperatures of T1 and T3 changed differently so that, while in the first 150 min, the temperature at T1 was higher than that of T3, the opposite took place in the remaining 150 min, such that the temperature of T3 exceeded that of T1, indicating that the heat transfer took place from location 1 to 2 by conduction through the water, which is minimised within the baffle region, so that the temperature of T4 becomes equal to that of T2 and T3. Overall, the difference between the four temperatures was narrower when the mirror inclination was 45° but, when equal to 80°, the difference was wider apart and T4 reached the highest temperature after 300 min of operation. It is recommended to use dual inclined mirror reflectors on both top sides of the SP at 45° to enhance the solar radiation during the diurnal period. It is worth mentioning that the reason for the aforementioned observation may be attributed to this EXP being carried out on a clear, sunny day, which proves that solar radiation is of prime importance in the performance of SPs.

### 3.2. Effect of Glass Wool

**Figure 3:**
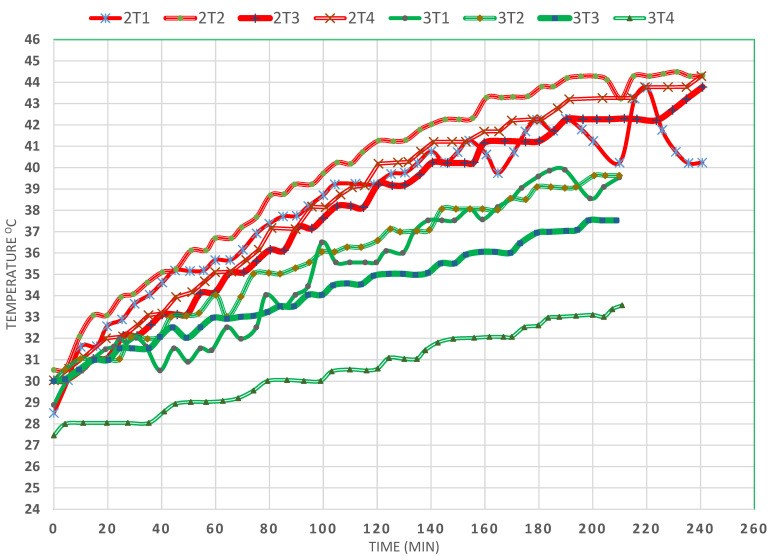
Effect of glass wool on the temperature profiles at various locations in the SP (EXPs (2) and (3)).

This factor can be observed by comparing EXPs (2) and (3), presented in Figure 3, from which it was observed that the only difference between the two is that, in EXP (3), 344 g of GW were used to fill the SP. It was thought that the convection currents might be suppressed by restricting the paths for the water flow by breaking them into small intertwining rivulets that are difficult to follow. According to expectations, and due to the reflective nature of GW, the T4 temperature increased only 7 °C within 240 min (4 h), whereas, in EXP (2), no GW was added, but the temperature of T4 increased about 14 °C, despite the fact that the mirror was used at an inclination equal to 80°; this was proven to be inefficient when comparing EXPs (1) and (2) for the effect of the mirror slope on the performance of the SP. As the figure shows, the T2 temperature was the highest in the first case among all the temperatures studied in the absence of the GW. Similarly, the temperature of T2 was the highest in the presence of the GW. The presence of the GW, moreover, led to a large ΔT between T2 and T3, followed by a larger ΔT between T3 and T4. This result was expected since most of the solar radiation was reflected by the lustrous nature of the GW and thus prevented the conduction of heat from below the baffles of T3 and T4 (the vertical midpoint in the coil region). Therefore, had the GW been black and matte, its effect would have been much different. This suggests future work in which somewhat similar packing to GW, but which is matte black, could be examined quickly, which could be achieved by painting the GW with black matte paint.

**Figure 4:**
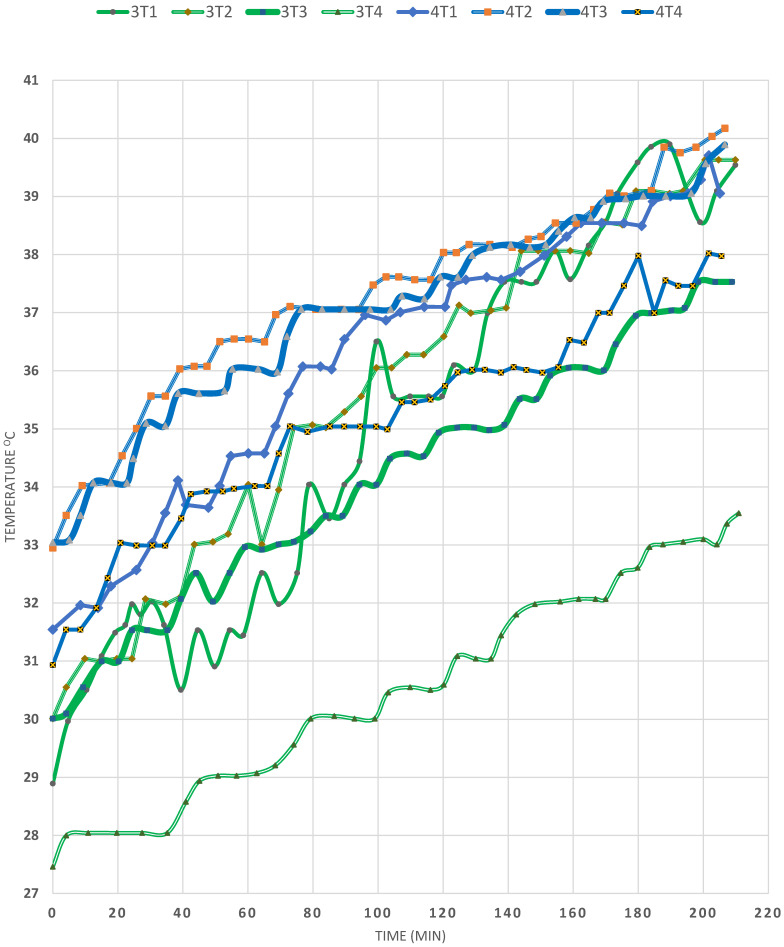
Effect of glass wool on the temperature profiles in the SP (EXPs (3)–(4)).

The effect of the quantity of the GW was also observed in Figure 4 by comparing EXPs (3) and (4). It is observed that T_a_ was lower in EXP (4) than EXP (3) by 4 or 5°C and that it was a windy day. On the other hand, some wind still prevailed during EXP (3); however, it was a sunny day. On comparing EXP (3) to (4), it is observed that T1 had the highest temperature in EXP (2), followed by that of T2, T3 and, least of all, T4, at the centre of the copper coil region, resulting from the presence of 344 g of GW inside the SP. Its effect was discussed in the aforementioned comparison between EXPs (2) and (3). However, in EXP (4) the quantity of GW was halved to 170 g; accordingly, the temperature profile inside the SP changed, so that the T2 temperature was the highest of all, increasing from 33 to 43 °C in 300 min of exposure to the solar radiation. Therefore, these results prove once more that GW is not recommended, since the temperatures of T1, T3 and T4 almost coincided during the 300 min of solar heating, which can be attributed to the fact that more heat transfer took place between the lower zones (T3 and T4), and that the T1 zone lost its heat to T2 due to the stagnant, nonconvective zone within the cruciform baffles. Thus, it could be stated that the baffles damped the convection currents, thereby causing the heat to be transferred from the surface downward to location 2. As a result, two findings were deduced from this study: first, that the cruciform baffles have a large effect on damping convection currents (the main reason that causes the destabilization of SPs); second, that GW—being specular to solar radiation—should be avoided since it causes large radiative heat losses, which makes the GW lose any benefit of damping the convection currents. It is noteworthy that, in the presence of either 344 or 170 g of GW, the T2 temperature increased about 8 °C in 180 min of solar energy storage.

### 3.3. Effect of Climatic Conditions

**Figure 5:**
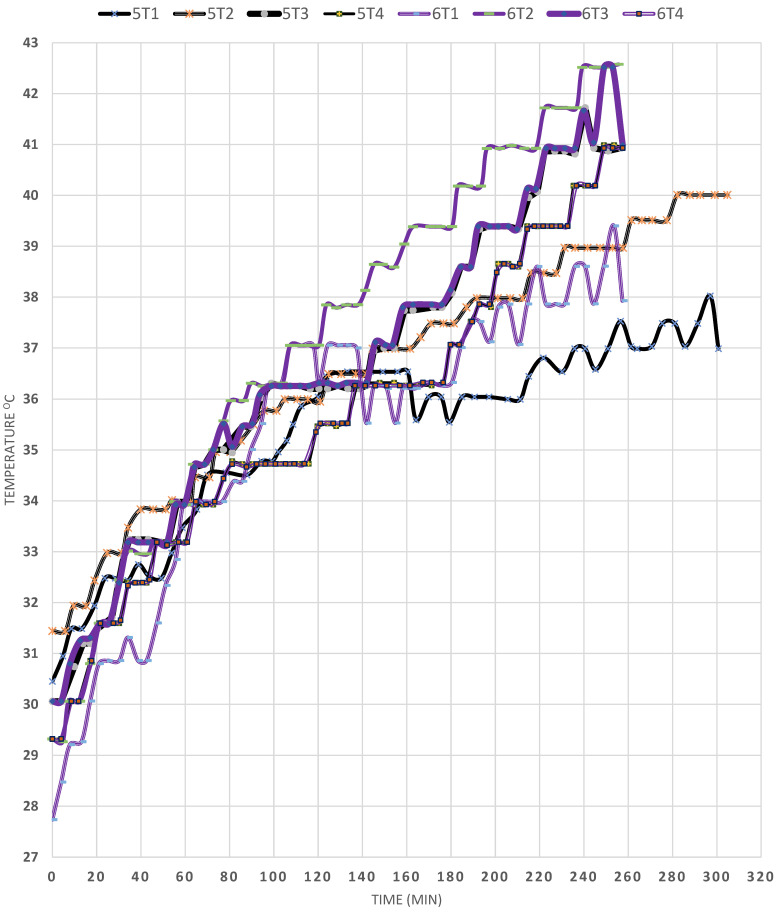
Effect of climatic conditions on the temperature profiles in the SP (EXPs (5) and (6)).

Examining EXPs (5) and (6) in Figure 5 and comparing them illustrates the influence of environmental circumstances on the performance of the SP. In EXPs (5) and (6), the T_a_ averages around 28.5 °C and 30 °C, respectively. However, EXP (5) was done on a windy day, but EXP (6) was undertaken on a calm day. Consequently, it was discovered that T1’s temperature rose from 30.5 °C to 36 °C during the course of 150 min, before reaching a total of around 37 °C. This result proves that the presence of wind results in heat loss from the SP, even though the GC hermetically seals the SP surface. As opposed to EXP (6), the T1 temperature increased from 29.5 °C to almost 41 °C (=11.5 °C), which is attributed to the absence of wind. However, it was observed that, in both cases, T2 was the highest among all the four temperatures, which indicates that the cruciform baffles have the beneficiary effect of damping the convection currents within the SP and assisting in the heat transfer by conduction, thus restricting heat loss. On the other hand, the T4 temperature followed that of T2, in the case of EXP (6), which proves that wind has a great deleterious effect on solar energy storage. In addition, the T4 temperature reached 39 °C and 41 °C in EXPs (5) and (6), in respective order; also, T3 (the temperature just below the baffles) was lower in the absence of wind, and was lower than the temperature of T4, in this case showing that the heat is transferred from T3 to T4 in EXP (6), as opposed to EXP (5), in which the T3 temperature was at all times higher than that of T4, meaning that the baffles were efficient in damping the convection currents, even below the baffle region.

**Figure 6:**
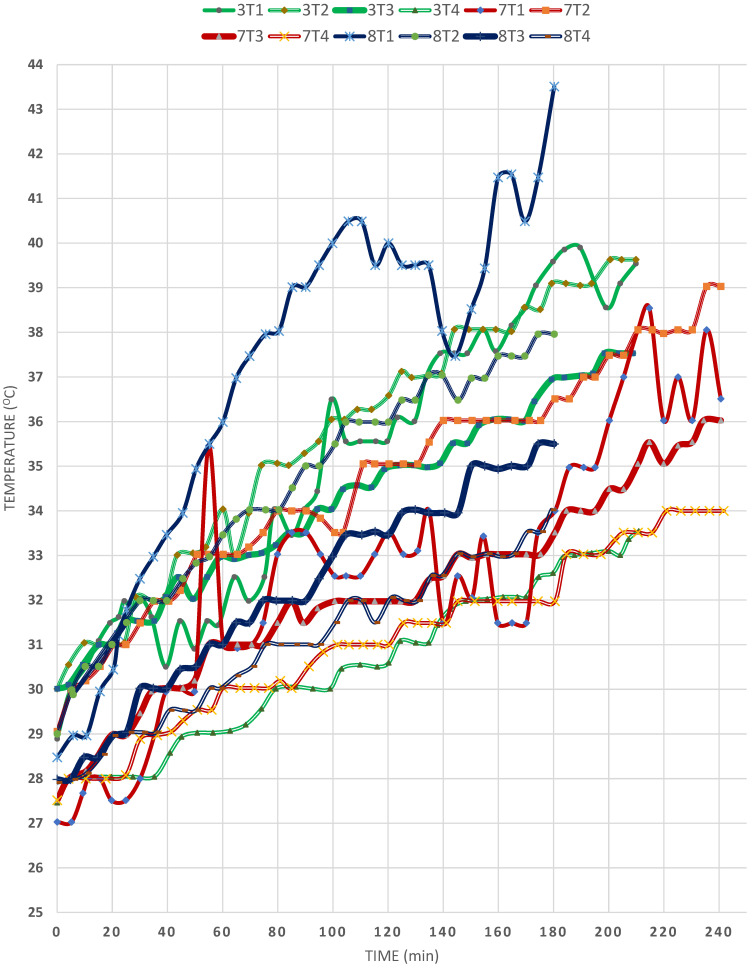
Effect of wind speed on the temperature profiles in the SP (EXPs 3, 7 and 8).

This factor is again reflected in comparing EXPs (3) and (7), in Figure 6, which were under similar climatic conditions as regards the solar radiation and ambient temperature, except that EXP (7) was conducted on a windy day. A glance at the two sets of curves shows that the T1 temperature was higher in the case of calm weather (EXP (3)), and the temperature overall was steadier than that in EXP (7), in which fierce winds prevailed. Moreover, the temperature in the case of EXP (3) reached a higher level after 250 min than that in the case of EXP (7), due to less heat loss by both convection and conduction. Regarding the temperature of T2, it is observed that, at the end of the experiment, it became higher in EXP 3 than in EXP 7, for the same reason. Furthermore, the T3 temperature was closer to that of T2 in the absence of wind, meaning that the loss of heat to the surroundings and the transfer of heat through conduction from the T2 zone to the T3 zone was greater in the case of EXP (3). On the other hand, the T4 zone had a similar pattern in both cases, which is expected, since in EXP (3) the conducted heat from the upper rim downward to position 4 caused the T2 zone to be heated easily, transferring some heat to the T3 zone and, finally, T4 but, in EXP (7), the temperature was confined within a 2–4 °C range only. However, it is noteworthy that the initial temperatures were slightly higher in the case of EXP (3), which must have partially contributed to these results. Overall, the wind is not recommended in the heating of SPs. However, so far, there are no techniques for its prevention.

The same factor can be studied by comparing EXP (3) to (8) (Figure 6), in which the latter was conducted on a windy day. It is observed that, in the case of EXP (5), the maximum temperature was T1 > T2 > T3 > T4, i.e., T4 was the maximum; also, the difference between the four temperatures was very narrow, which emphasised the deleterious effect the wind has on heating SPs. Oppositely, EXP (6), which was discussed earlier, proves that the operation is steadier and more worthwhile.

### 3.4. Reproducibility

**Figure 7:**
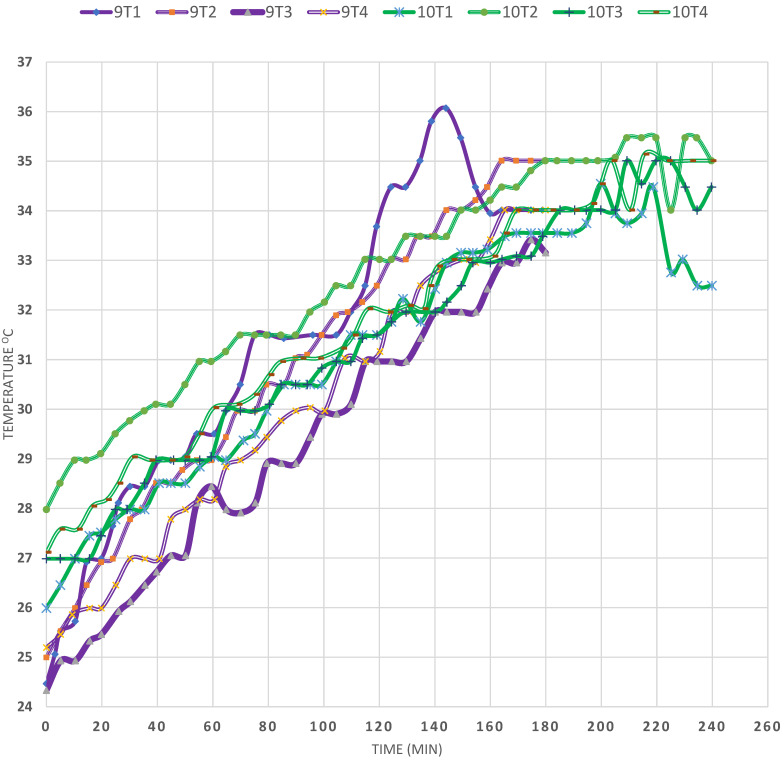
Effect of reproducibility (EXPs 9 and 10).

On comparing EXPs (9) and (10), in Figure 7 to each other, and in which the conditions were almost identical, it is observed that the temperature of T2 was almost equal in the two EXPs, reaching almost 35 °C after 180 min of solar heating. In addition, the T3 temperature was about 2 °C lower than that of T2, in both cases. Moreover, it is clear that all the temperatures in both experiments ranged between 34 °C and 35 °C after the lapse of about 225 min, i.e., they almost overlap. These observations reveal the consistency of the results, as well as their reproducibility. Nevertheless, it must be mentioned that solar heat storage in SPs depends on several factors simultaneously, since they are inseparable.

### 3.5. Effect of the Presence of Thickening Material

**Figure 8:**
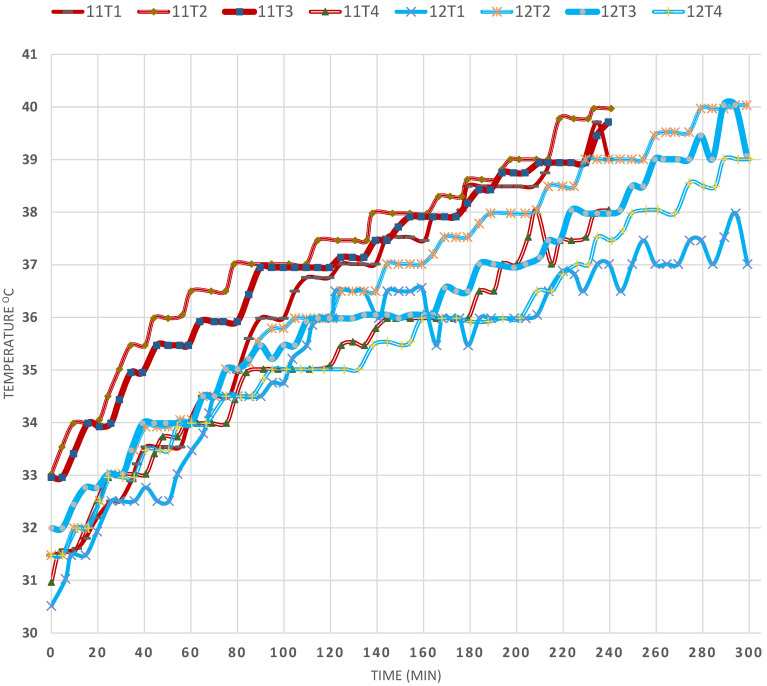
Effect of gel thickening material on the temperature profiles in the SP (EXPs 11 and 12).

This factor was studied by comparing EXPs (11) and (12), in Figure 8, in which the latter contained a thickening material (TM) to damp the convection currents due to increased viscosity, so that the heat transfer would take place between the water layers by conduction. It was observed that, in EXP (11), in which no additive was added, the T2 temperature increased from 33 °C to 40 °C in 300 min whereas, in the presence of the thickening agent, the T2 temperature increased from 31 °C to 40 °C. These results prove that increasing the viscosity stabilised the SP and minimised the convection currents, together with the baffles. However, the T4 temperature in the absence of Tm was substantially lower than that of T2 and T3, whereas, in the presence of (TM), the difference in temperatures was less, due to the damping of the convection currents and, even after 300 min of heating, the T4 temperature was only 1 °C lower than that of T2. However, the temperature of T3 was between that of T2 and T4 in both cases after 300 min of operation.

### 3.6. Effect of Ambient Temperature

**Figure 9:**
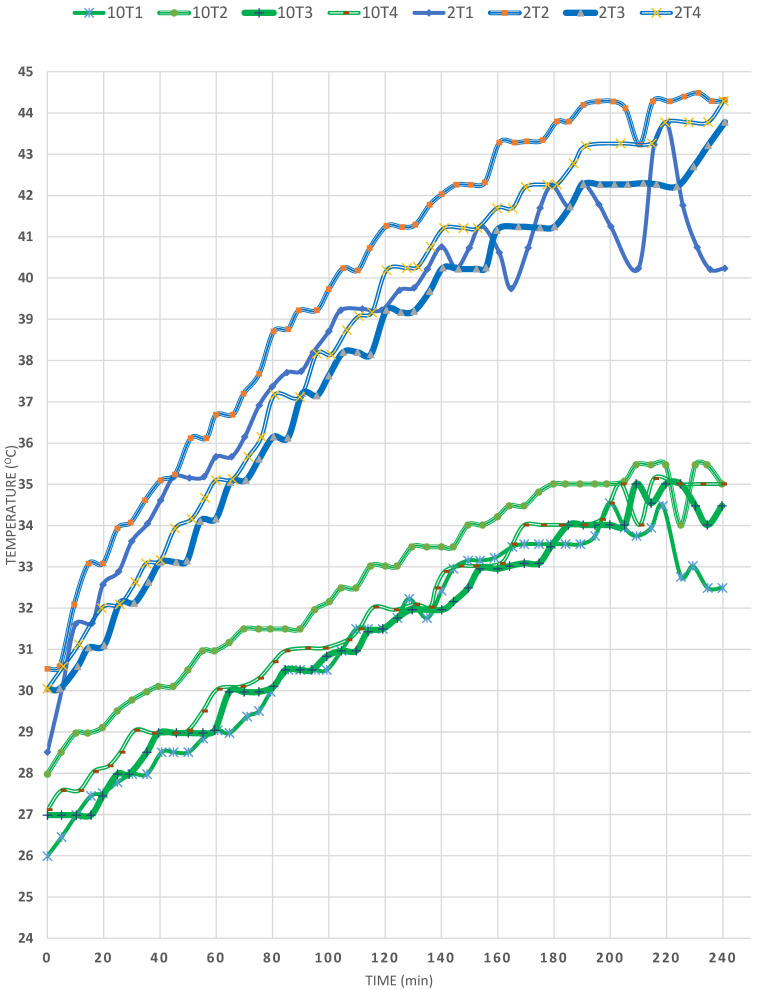
Effect of ambient temperature in the SP (EXPs (2) and (10)).

The effect of ambient temperature is manifested by comparing EXP (2) to EXP (10) in Figure 9, in which T_a_ was 30 °C in both experiments. It is observed that, for 4 h, the T1 temperature increased from 26 °C to about 33 °C in EXP (10) whereas, in EXP (2), the temperature increased from 28 °C up to 44 °C, then decreased to 40 °C in 4 h of solar heating. The significant difference in the maximum T obtained between the two cases is therefore around 10 °C, which is attributed to the much higher T_a_ in the second experiment. However, the highest temperature obtained in both cases was that of T2 (44.5 °C and 35 °C in EXP (2) and (10), in respective order), which once again proves that the baffles damped the occurrence of convection currents in both cases; yet, still ΔT2 between EXP (10) and (2) is a high value of 9 °C, as expected. As to the temperature of T4, it is clear from the figures that it was right below that of T2 in both experiments, but the difference between T2 and T4 was larger in the first case and only about 1 °C different in EXP (2). This was also expected, since the conditions are more optimum in EXP (2), where the maximum ambient temperature attained was 27 °C. In EXP (10), ΔT was about 2 °C, thus T_a_ is found to affect the temperatures at all locations in the SP due to the reduction of heat loss from the SP, both by radiation to the surroundings and convection currents to other lower temperatures as found in the T3 and T1 zones. One more observation is that the lowest temperature in the SP after 4 h of solar heating was 34 °C and 44 °C of T3 in EXPs (10) and (2), respectively, which still indicates that the temperature of T4 has increased above T3 in both cases. The increase is recognized in EXP (2) rather than in EXP (10), which shows that, at the end of 4 h of heating, the temperature of T4, in the region of the copper coils, becomes higher than that of T3 (just below the cruciform baffles), which proves again that the baffles lead to high heat transfer from the top downwards, so that the maximum heat is achieved at the zone wherein use is made of the heat in heating the flowing water inside the copper coil, which, in turn, is to be made use of in heating any facility outside the SP. On the other hand, these results were obtained even though that the mirror slope was 45° in EXP (10). In EXP (2), the slope of 45° was found to lead to better heating, as shown in previous discussions. Overall, the previous discussions illustrate the immense importance of the climatic conditions, as regards T_a_ and the wind speed. Table 2 presents the determination coefficients and differences in temperatures for each temperature profile for both EXPs.

### 3.7. Thermal Efficiency of the Pond

The percentages of the maximum thermal saltless SP efficiency for all the carried-out experiments are presented in Table 3. At an ambient temperature of 30 °C, on a calm sunny day with a wind speed of 7 km/h, the best thermal storage efficiency of 32.58 per cent was obtained in the presence of the cruciform baffles, with a 45° slant of a mirror fixed to the top rim of the SP (EXP (2)). The lowest percentage efficiency obtained was 8.82 per cent at an ambient temperature of 31 °C, on a windy sunny day with a wind speed of 26 km/h, with an 80° inclination of the mirror fixed to the top rim of the SP and 344 g of GW (EXP (8)). It is worth noting that the presence of the thickening material enhanced the pond efficiency by more than 2% (EXPs (11) and (12)).

## 4. Conclusions and Recommendations

Solar ponds have been constructed in large areas from salty water, in which the salt concentration increases from the surface vertically downwards to the bottom to avoid mixing due to convection currents. Alternatively, saltless SPs have been studied lately to prevent limitations caused by the water’s salinity, such as corrosion problems, etc. In the present work, a miniature, saltless SP was constructed in which cruciform baffles were used to damp the convection currents, which are the main cause of an SP losing its functionality for long-term solar thermal energy storage. The SP is composed of two sections in which, instead of making the salinity increase steadily from the surface to the bottom, the UNCZ is provided with baffles running from the surface to the middle of the SP, while the LCZ contains a copper coil within which cold water can be heated by the exchange of heat from the lower water layers in the LCZ, then pumped to the outside for the provision of heat in a heat exchanger. The following conclusions were arrived at: firstly, the saltless SP holds more heat between sunrise and sunset than the SGSP. Fitting transparent cruciform baffles prevents convection currents and thus can replace the addition of salts to achieve stabilization of the water in the pond, allowing the temperature to increase steadily from top to bottom. It was also found that the addition of a gelling material (preferred are paraffin oil or any non-toxic, environmentally acceptable liquid that is clear, chemically stable when exposed to sun radiation, and inexpensive) to the pond increased the water viscosity and thus damped the heat loss due to the convection currents. Also, the addition of GW did not improve the performance of the SP, due to its light-scattering properties, and its addition did not realise the intended effect of making the path of water between the GW fibres slow down, thereby minimizing the deleterious effect of the convection currents. Another conclusion is that placing a mirror at a 45° slope could be beneficial in providing the proper tilt to the horizontal to reflect the solar radiation onto the pond’s surface. Finally, as expected, the climatic conditions, particularly the prevailing wind, affect the performance of the SP adversely; however, this factor cannot be avoided. The temperature of the pond is determined mostly by the heat loss through the pond’s upper surface and the shadowing of the incident solar radiation. The quick increase in temperature of the pond water makes saltless SPs more appropriate for short-term energy storage.

The potential applications of such saltless SPs include being installed on the rooftops of buildings, resulting in a significant cost decrease in comparison to a typical metallic collector with a heat tank. Due to the modest surface area of such a solar system, the potential environmental issues associated with the usage of gelling material are minimal. In addition, the yearly maintenance costs for such a system are minimal.

The development of a hemispherical structure to reduce shading, effective turbidity control techniques, effective heat extraction methods based on nanofluids, efficient hybrid solar pond integration with a solar collector, air conditioning, solar chimney, desalination, and power generation systems may be among the areas of solar pond research to be pursued in the future.

## Figures and Tables

**Table 1:** Conditions and variables investigated throughout the experiments.

EXP No.	Variables Investigated						
	Ambient Temperature, Ta, °C	Presence or Absence of Baffles	Presence of GC	Slope of the Mirror at the Top of SP	Presence of GW Inside SP and Its Quantity, g	Wind Speed, km/h	Presence of Thickening Gel Material
1	31.5	Yes	Yes	80°	No	3–7	No
2	30	Yes	Yes	45°	No	7	No
3	30.5	Yes	Yes	80°	344	11	No
4	26.5	Yes	Yes	No	170	16	No
5	28.5	Yes	Yes	45°	No	21	No
6	30	Yes	Yes	45°	No	3–7	No
7	30	Yes	Yes	80°	344	24	No
8	31	Yes	Yes	80°	344	26	No
9	29.5	Yes	Yes	45°	No	13	No
10	30	Yes	Yes	45°	No	12	No
11	32	Yes	Yes	No	No	3–7	No
12	32	Yes	Yes	No	No	3–7	Yes

**Table 2:** Thermal coefficient for temperature profile data of Figure 6.

EXP No.	Ambient Temp., T_a,_ °C	T1		ΔT1	R^2^	T2		ΔT2	R^2^	T3		ΔT3	R^2^	T4		ΔT4	R^2^
		Start	End			Start	End			Start	End			Start	End		
2	30	28.5	40.2	11.7	0.964	30.5	44.5	14	0.994	30	43.7	13.7	0.994	30	44.5	14.5	0.996
10	30	26	32.5	6.5	0.961	28	35	7	0.982	27	34.5	7.5	0.991	27	35	8	0.987

**Table 3:** % Maximum thermal pond efficiencies for carried-out experiments according to conditions of Table 2.

EXP No.	Ambient Temperature, T_a_, °C	LCZ Temperature	% Max Thermal Pond Efficiency
1	31.5	43.3	27.25
2	30	44.5	32.58
3	30.5	33.5	8.96
4	26.5	38	30.26
5	28.5	41	30.49
6	30	41	26.83
7	30	34	11.76
8	31	34	8.82
9	29.5	34	13.24
10	30	35	14.29
11	32	38	15.79
12	32	39	17.95

## Data Availability

Not applicable.
